# Characterization of the whole chloroplast genome of *Chikusichloa mutica* and its comparison with other rice tribe (Oryzeae) species

**DOI:** 10.1371/journal.pone.0177553

**Published:** 2017-05-24

**Authors:** Zhiqiang Wu, Cuihua Gu, Luke R. Tembrock, Dong Zhang, Song Ge

**Affiliations:** 1 State Key Laboratory of Systematic and Evolutionary Botany, Institute of Botany, Chinese Academy of Sciences, Beijing, China; 2 School of Landscape and Architecture, Zhejiang Agriculture and Forestry University, Hangzhou, China; 3 Department of Biology, Colorado State University, Fort Collins, Colorado, United States of America; 4 Department of Statistics, Iowa State University, Ames, Iowa, United States of America; The National Orchid Conservation Center of China; The Orchid Conservation & Research Center of Shenzhen, CHINA

## Abstract

Chloroplast genomes are a significant genomic resource in plant species and have been used in many research areas. The complete genomic information from wild crop species could supply a valuable genetic reservoir for breeding. *Chikusichloa mutica* is one of the most important wild distant relatives of cultivated rice. In this study, we sequenced and characterized its complete chloroplast (cp) genome and compared it with other species in the same tribe. The whole cp genome sequence is 136,603 bp in size and exhibits a typical quadripartite structure with large and small single-copy regions (LSC, 82,327 bp; SSC, 12,598 bp) separated by a pair of 20,839-bp inverted repeats (IR_A, B_). A total of 110 unique genes are annotated, including 76 protein-coding genes, 4 ribosomal RNA genes and 30 tRNA genes. The genome structure, gene order, GC content, and other features are similar to those of other angiosperm cp genomes. When comparing the cp genomes between Oryzinae and Zizaniinae subtribes, the main differences were found between the junction regions and distribution of simple sequence repeats (SSRs). In comparing the two *Chikusichloa* species, the genomes were only 40 bp different in length and 108 polymorphic sites, including 83 single nucleotide substitutions (SNPs) and 25 insertion-deletions (Indels), were found between the whole cp genomes. The complete cp genome of *C*. *mutica* will be an important genetic tool for future breeding programs and understanding the evolution of wild rice relatives.

## Introduction

The grass family (Poaceae) is one of the most diverse angiosperm families and contains numerous economically important crop species [1]Grass Phylogeny Work. Group II. 2012), including rice (*Oryza sativa*), the most economically important species in the world [2]. Because of its economic value, this species and even the *Oryza* genus has been used as a model system to conduct numerous genetic and evolutionary studies [3, 4]. The rice (*Oryza*) species and its many wild relatives are categorized into two well-supported subtribes, Oryzinae and Zizaniinae, in the subfamily Ehrhartoideae [5, 6]. In each subtribe, many species have economic value and have been used as food for many centuries, such as the two main cultivated rice species (*Oryza sativa* and *O*. *glaberrima*) in Oryzinae [7] and the wild rice species *Zizania latifolia* and *Z*. *aquatica* in Zizaniinae [8]. In addition to these species, many wild relatives in the Oryzeae tribe possess enormously useful genetic resources for improving rice breeding through increasing yields [9] and providing tolerance from environmental stress [10]. While the species in the Oryzinae tribe have been studied in depth with regard to their genetic importance [2, 11, 12, 13], the species in Zizaniinae have not been as thoroughly examined, except for the organelle genomes [14, 15, 16]. *Chikusichloa* is one such example of a genus from Zizaniinae for which we have only limited knowledge regarding the chloroplast genome. *Chikusichloa* is only made up of three perennial species in Southeast Asia, which are all uncommon within their range. The range of *Chikusichloa* extends from Indonesia (Sumatra) in the south to Japan and China in the north. The habitat of *Chikusichloa* includes wet swampy areas amid forests. *C aquatica* Koidz grows in wet valleys and on stream sides in China and Japan; *C*. *mutica* Keng is found in damp stream sides in forests of China and Indonesia; and *C*. *brachyathera* Ohwi is only found in the Ryukyu Islands [17]. Completion of their organelle genomes would supply a rich repository of genetic material for future breeding programs.

Chloroplasts, which are the photosynthesis organelle in plant and algae cells, originated from cyanobacteria through endosymbiosis approximately one billion years ago [18] and retained their own genome through uniparental inheritance [19]. Many essential metabolites are synthesized in chloroplasts, such as fatty acids, starch, pigments, and amino acids [20]. Over time, chloroplast genomes have experienced dramatic variation, but a conserved structure has been maintained within land plants. The chloroplast genome structure is characterized by a small genome size with a circular quadripartite structure ranging from 120–165 kb in length, containing a pair of inverted repeats (IRs) separated by a large single-copy region (LSC) and a small single-copy region (SSC) [21, 22]. With the development of high throughput sequencing technologies [23] and the conserved features of chloroplast genomes [21, 24], over 1,000 species in Viridiplantae have been completely sequenced and published in the NCBI Organelle Genome Resources database (http://www.ncbi.nlm.nih.gov/genome/organelle/). The highly conserved gene order, stable gene content, and slow rate of mutation in chloroplast genomes [24, 25, 26] have made them an important genetic resource to explore evolutionary variation in land plants. For example, dozens of molecular markers or even the whole chloroplast genome have been used for plant molecular systematic and taxonomic studies [27, 28] in the field of plant biogeography [29] and for DNA barcoding [30]. In addition, using chloroplasts in genetic engineering also offers certain unique advantages over nuclear genomes, including high transgene expression [31, 32] and the containment of transgenes through maternal inheritance [33]. Thus, it is a valuable genetic resource to complete the chloroplast genomes from wild rice relatives.

In this study, by employing traditional Sanger sequencing and sets of conserved universal primers from grass species, we assembled a high quality complete chloroplast genome of *Chikusichloa mutica* and deposited the annotated sequence into the NCBI database. We also conducted a comprehensive comparison with the other published chloroplast genome of *C*. *aquatica* (KR078265) [16] to detect all polymorphisms between the two whole chloroplast genomes. Utilizing the whole chloroplast, we reconstructed the phylogenetic relationships of all rice tribe species and compared their genomic features and structural variation.

## Material and methods

### Complete chloroplast genome of *Chikusichloa mutica*

Fresh leaves of the *Chikusichloa mutica* were collected from a plant (originally collected in the wild by Prof. Song Ge #GS0601 for [34]) grown in the greenhouse of the Institute of Botany of the Chinese Academy of Sciences in Beijing. The total cellular DNA was extracted using the cetyltrimethyl ammonium bromide (CTAB) method and purified with phenol extraction [34]. Amplification and Sanger sequencing methods were employed to complete the whole chloroplast genome of *C*. *mutica*. Based on the conserved features of chloroplast genome in land plants [21, 24] and our previous result [14, 15], by using the chloroplast primers from Wu et al [35], we successfully amplified the entire chloroplast in overlapping fragments. Conditions for PCR amplification were 4 min of initial denaturation at 94°C, 35 cycles of 45 s at 94°C, 45 s annealing at 52°C, and 90 s extension at 72°C, followed by a final 10-min incubation at 72°C. The PCR products were purified as described in Tang et al [34] and directly sequenced on an ABI 3730 (Applied Biosystems, Foster City, CA, USA). The final Sanger sequences were trimmed and assembled with the ContigExpress program from the Vector NTI Suite 6.0 (Informax Inc., North Bethesda, MD).

### Chloroplast genome annotation

**Fig 1:**
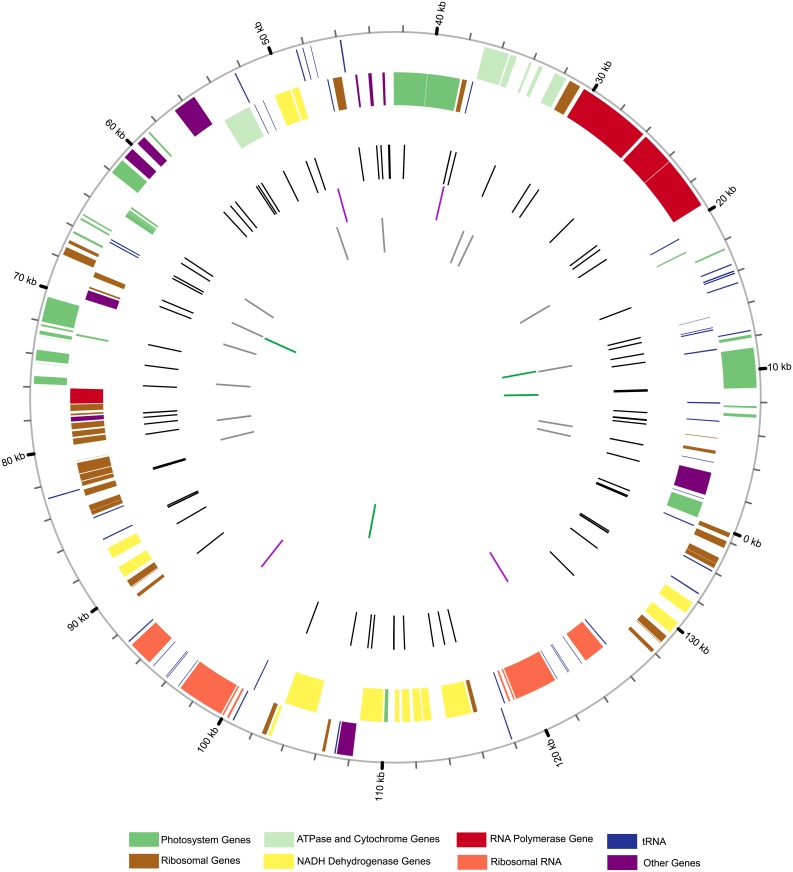
The simplified schematic diagram showing the chloroplast genome information and variation maps of *Chikusichloa mutica*. From outside to inside, all tracks independently represent: 1) the forward strand coding genes; 2) the reverse strand coding genes; 3) the number and distribution of single nucleotide substitutions (SNPs) (black bar color); 4) the number and distribution of non-repeat insertion-deletions (Indels) (purple bar color); 5) the number and distribution of homopolymer structures (grey bar color); 6) the number and distribution of repeat Indels (green bar color). The different functional groups of chloroplast coding genes are colored at the bottom. The diagram was generated with Circos v0.67 (http://circos.ca/).

The final assembled chloroplast sequence was submitted to DOGMA (Dual Organellar GenoMe Annotator, http://dogma.ccbb.utexas.edu/) for annotation. The original DOGMA draft output contained many errors caused by variation of the exon–intron boundaries of genes or the questionable positioning of the start and stop codons. To finish the final annotation, we subsequently inspected all the inaccurate positions and performed blast searches within the published chloroplast genome database of related species to perform manual adjustments. Both tRNA and rRNA genes were identified by combining the BLASTN searches with relative species in rice tribes [14] and the DOGMA tools. The final annotation was submitted to GenBank and the diagrammatic annotation of the chloroplast genome was plotted using the bioinformatics tools in Circos 0.67 [36] (Fig 1).

### Polymorphisms detection

To compare the polymorphisms in detail between the whole chloroplast genomes within *Chikusichloa*, the published genome data from *C*. *aquatica* (KR078265) [16] was employed for comparison with our newly completed chloroplast genome of *C*. *mutica*. Based on the conserved structure of chloroplast genomes within the grass family [14, 37], the two genome sequences could be aligned by synteny. MAFFT v7.221 [38] was used to conduct the whole chloroplast genome alignment under the FFT-NS-2 setting, followed by manual adjustment. The two aligned genome sequences were used to extract the number and position of the polymorphic sites by DnaSP v5.10 [39], including the SNPs (single nucleotide polymorphisms) and Indels (insertion/deletions).

### Simple sequence repeats (SSRs)

Simple sequence repeats (SSRs), also known as microsatellites with 1–6 bp long repeat motifs, are common genomic features, with high rates of polymorphism due to their slip strand mis-pairing mutation mechanism [40]. They have been widely used as co-dominant molecular markers in marker assisted breeding, population genetics, and genetic linkage mapping [41]. To identify the distribution of SSRs across the chloroplast genome, the public Perl script MISA (http://pgrc.ipk-gatersleben.de/misa/) was employed. The identification of SSRs included motif sizes from one to six nucleotide units with repeat lower thresholds set to of 6, 5, 4, 3, 3, and 3 repeat units for mono-, di-, tri-, tetra-, penta-, and hexa-nucleotide SSRs, respectively. *Chikusichloa mutica* and 13 other species in the rice tribe were examined for SSRs. *Potamophila parviflora* (GU592210) and *Microlaena stipoides* (GU592211) were excluded from this analysis due to their incomplete chloroplast genomes.

### Chloroplast phylogenomics analysis

As an important target in plant systematics, the chloroplast genome has been widely used to resolve phylogenetic relationships among plant lineages [19]. To further determine and validate the phylogenic relationships of *C*. *mutica* with other Oryzeae species, published chloroplast genomes were included in the phylogenetic analysis, including 15 species from the subfamily Ehrhartoideae (Table 1) and one species (*Phyllostachys propinqua*) from Bambusoideae. A total of 17 species’ whole chloroplast genome data were included in the phylogenetic analysis. The complete chloroplast genome alignment from 17 species was used to construct the phylogenetic tree based on the conserved structure among grass family chloroplasts [14, 37, 42]. The alignment employed MAFFT v7.221 [38] using the same settings as mentioned in the annotation section above. The final alignments (S1 File) were used to resolve relationships using three different phylogenetic-inference methods: maximum parsimony (MP) analysis in PAUP* 4.0b10 [43]; Bayesian inference (BI) in MrBayes 3.1.2 [44] and maximum likelihood (ML) with PHYML Version 2.4.5[45] applying the settings mentioned previously [14].

**Table 1:** Base composition in various regions of the *Chikusichloa mutica* chloroplast genome.

Regions	A%	T%	C%	G%	GC%	Length (bp)
Total	30.63	30.34	19.44	19.60	39.04	136,603
LSC	31.25	31.54	18.38	18.82	37.20	82,327
SSC	35.84	30.79	17.25	16.12	33.37	12,598
IR _(A,B)_	27.73	27.91	21.29	23.08	44.37	20,839
CDS^a^	29.39	31.16	18.27	21.18	39.45	55,521
1st	28.97	23.28	19.01	28.74	47.75	18,507
2nd	27.51	32.92	21.10	18.47	39.57	18,507
1st+2nd	28.24	28.10	20.06	23.60	43.66	37,014
3rd	31.69	37.27	14.71	16.33	31.04	18,507

LSC: large single-copy region; SSC: small single-copy region; IR: inverted repeat; CDS: protein-coding region.

^a^: if some genes have two copies, only one copy is included.

## Results

### Genome assembly and feature

By employing the full set of the primers from Wu et al [35], the complete chloroplast genome of *C*. *mutica* was sequenced and assembled. For each amplicon, we conducted bi-directional Sanger sequencing to obtain high-quality sequencing bases. After assembly and editing, the whole chloroplast genome sequence was 136,603 bp in length. The genome was annotated following the methods of Wu and Ge [14] and deposited into GenBank with accession number KU696970.

The chloroplast genome of *C*. *mutica* is a typical quadripartite structure consisting of a pair of inverted repeats (IRs) with a length of 20,839 bp separated by a small single-copy region (SSC) of 12,598 bp and a large single-copy region (LSC) of 82,327 bp, respectively (Fig 1; S1 Fig; Table 1). It is a AT-rich genome typical of most land plants [18] with a GC content of only 39.04%, similar to most of the published chloroplast genomes in the rice tribe (Table 2). The GC content of the two IR regions was 44.37%, which is higher than 37.20% of the LSC region and 33.37% of the SSC region (Table 1). The higher GC content of the IR regions was due to the high (54.78%) GC content of the four ribosomal RNAs (rRNAs). The overall average GC content of the rice tribe species was 38.99% (±0.0004), with the highest GC content in the IR region (44.34%) and the lowest in the SSC region (33.31%) (Table 2).

**Table 2:** Comparison of major features of 18 Poaceae chloroplast genomes from Ehrhartoideae and Bambusoideae subfamilies.

Subfamily	Tribe (Subtribe)	Species	Total size		LSC region		IR region		SSC region		GenBank Accession
			Length (bp)	GC (%)	Length (bp)	GC (%)	Length (bp)	GC (%)	Length (bp)	GC (%)	
Ehrhartoideae	Oryzeae (Oryzinae)	*Oryza sativa* ssp. *indica*	134,496	39.00	80,553	37.11	20,798	44.35	12,347	33.32	NC_008155
		*Oryza sativa* ssp. *japonica*	134,551	39.00	80,604	37.11	20,802	44.35	12,343	33.37	AY522330
		*Oryza nivara*	134,494	39.01	80,544	37.12	20,802	44.35	12,346	33.33	NC_005973
		*Oryza barthii*	134,674	38.99	80,685	37.10	20,804	44.34	12,381	33.33	NC_027460
		*Oryza glumipatula*	134,583	38.99	80,613	37.09	20,807	44.34	12,356	33.32	NC_027461
		*Oryza punctata*	134,911	39.00	80,955	37.10	20,813	44.36	12,330	33.37	NC_027676
		*Oryza officinalis*	134,604	38.97	80,623	37.08	20,797	44.35	12,387	33.28	NC_027463
		*Oryza australiensis*	135,224	38.95	81,074	37.07	20,840	44.33	12,470	33.18	KJ830774
		*Oryza brachyantha*	134,604	38.98	80,411	37.10	20,832	44.31	12,529	33.31	KT992850
		*Leersia tisserantii*	136,550	38.88	81,865	37.01	21,329	44.05	12,027	33.23	JN415112
	Oryzeae (Zizaniinae)	*Zizania latifolia*	136,461	39.00	82,115	37.13	20,878	44.42	12,590	33.18	KT161956
		*Zizania aquatica*	136,364	39.02	82,013	37.14	20,879	44.41	12,593	33.31	KJ870999
		*Rhynchoryza subulata*	136,303	39.00	82,029	37.14	20,840	44.36	12,594	33.40	JN415114
		*Chikusichloa aquatica*	136,563	39.04	82,314	37.21	20,838	44.37	12,573	33.41	KR078265
		***Chikusichloa mutica***	136,603	39.04	82,327	37.20	20,839	44.37	12,598	33.37	**KU696970**^a^
		*Potamophila parviflora*	134,551	39.07	80,604	37.19	20,800	44.32	12,347	33.58	GU592210^b^
	Ehrharteae	*Microlaena stipoides*	134,551	39.22	80,613	37.28	20,793	44.18	12,343	33.77	GU592211^b^
Bambusoideae	Bambusodae	*Phyllostachys propinqua*	139,704	38.88	83,227	36.96	21,800	44.23	12,877	33.14	JN415113

^a^ Sequenced in this study;

^b^ unfinished chloroplast genome.

To understand the structural differences between chloroplasts in the rice tribe, we compared 15 genomes in the rice tribe and one from bamboo (Table 2). The total length variation between the complete genomes was approximately 2 kb, ranging in length from 134,494 bp to 136,603 bp with the species in Zizaniinae longer than in Oryzinae. The main contribution to the difference in length is found in the LSC regions, with lengths ranging from 80,411 bp to 82,327 bp (Table 2). The other regions, including the two IR and SSC regions, are relatively conserved in length within the rice tribe.

It has been shown that chloroplast genomes are conserved in gene content and gene order across the grass family [46]. For the final annotation, we predicted a total of 128 functional genes in the chloroplast genome of *C*. *mutica* with 110 unique genes and 18 duplicated genes in the IR regions (Fig 1, S1 Table). Among the 110 unique genes, 76 were protein-coding genes and 34 were RNA genes, including 30 tRNA genes and four rRNA genes (S1 Table). For the 18 duplicated genes in the IR regions, there were six protein-coding genes, eight tRNA genes, and four rRNA genes (S1 Table). Sixteen genes contained introns; 14 contained a single intron (eight protein-coding and six tRNA genes) and *ycf3* contained two introns. The *rps12* gene was found to be trans-spliced with the 5′end exon located in the LSC region and the two 3′end exons duplicated in the IR region. The *trnK*-UUU gene had the largest intron (2,487 bp) with the gene *matK* located within this intronic region. The total length of 76 protein-coding genes was 55,521 bp, and the GC content for the first, second, and third codon positions was 47.75%, 39.57%, and 31.04%, respectively (Table 1). The lower percentage of GC nucleotides in our dataset at the third codon position corresponds to previous findings in which the third codon positions are AT-biased in the chloroplasts of land plants.

### Simple sequence repeats (SSRs)

SSR markers have been widely used in plant genetics studies and will constitute an important genomic resource with the development of NGS (Next Generation Sequencing) technologies [41]. In this study, we identified a total of 133 SSR loci, including 115 mono-nucleotides, four dinucleotides, three tri-nucleotides, ten tetra-nucleotides, and one penta-nucleotide (Table 3) from the whole chloroplast genome of *C*. *mutica*. The majority of the SSR loci were mononucleotides (86.47%), and of those, 91.30% were A/T motifs. These analyses demonstrate that the SSRs in chloroplast genomes are commonly composed of polyadenine (polyA) or polythymine (polyT) repeats [47]. In addition to SSR identification, we also conducted a comparative analysis across chloroplast SSRs in the rice tribe (Table 3). The main source of length variation came from mononucleotide SSRs, in which Zizaniinae chloroplasts possessed more than 110 mononucleotide SSRs of eight nucleotides long or longer and the Oryzinae species sampled possessed fewer than 100 such SSRs. All other SSR motifs were at the same length across the examined chloroplasts among all species.

**Table 3:** Comparison of the number of SSRs of 14 chloroplast genomes from rice tribe.

Species	mono-nucleotide 6 units (8 units)	di-nucleotide (5 units)	tri-nucleotide (4 units)	tetra-nucleotide (3 units)	penta-nucleotide (3 units)	hexa-nucleotide (3 units)	Total
*Oryza sativa* ssp. *Japonica*	511 (89)	4	3	8	0	1	527 (105)
*Oryza nivara*	509 (85)	4	3	9	1	0	526 (102)
*Oryza barthii*	511 (87)	4	3	9	0	2	529 (105)
*Oryza glumipatula*	509 (87)	4	3	9	0	0	525 (103)
*Oryza punctata*	497 (91)	4	3	10	0	0	514 (108)
*Oryza officinalis*	500 (93)	5	3	9	1	0	518 (111)
*Oryza australiensis*	500 (94)	4	4	9	0	0	517 (111)
*Oryza brachyantha*	514 (89)	3	3	7	0	0	527 (102)
*Leersia tisserantii*	505 (100)	2	1	9	2	0	519 (114)
*Rhynchoryza subulata*	509 (111)	5	2	8	0	0	524 (126)
*Zizania latifolia*	509 (111)	3	4	10	1	1	528 (130)
*Zizania aquatica*	515 (116)	3	3	9	2	0	532 (133)
*Chikusichloa aquatica*	497 (113)	4	3	10	1	0	515 (131)
***Chikusichloa mutica***	**503 (115)**	**4**	**3**	**10**	**1**	**0**	521 (133)

### Dynamic variation of the junctions

**Fig 2:**
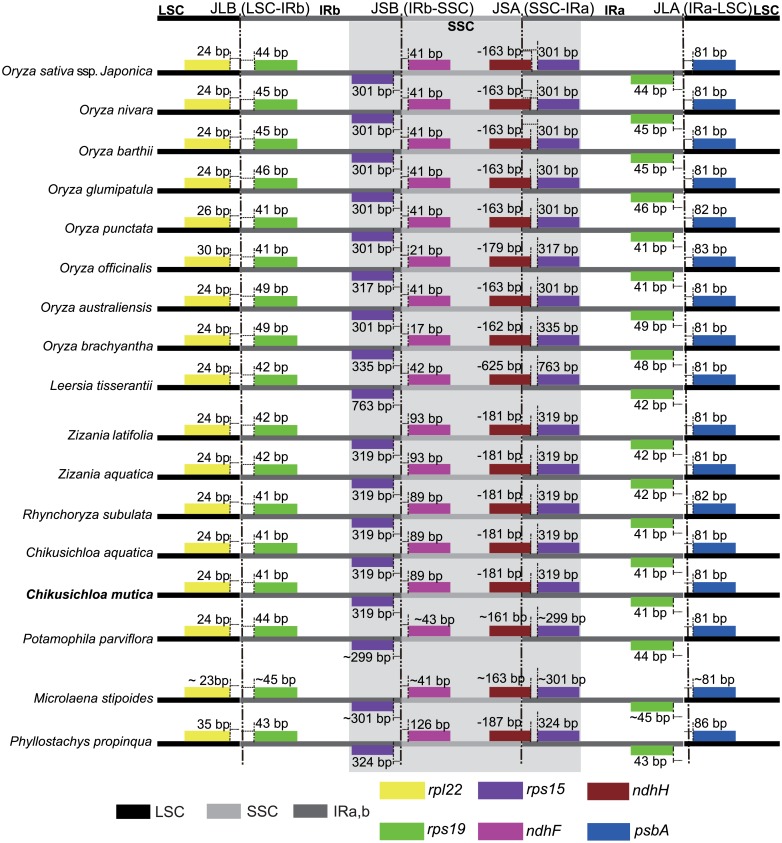
The variations of border distances between adjacent genes and four junction regions among 16 grasses’ chloroplast genomes. Boxes above or below the main line indicate the adjacent border genes, which were represented by the different colored boxes at the bottom. The LSC, SSC and two IR regions were also color coded. The distance is not scaled with sequence length.

The typical quadripartite structure of chloroplast genome possesses four junctions (J_LA_, J_LB_, J_SA_, and J_SB_) between the two IRs (IR_A_ and IR_B_) and the two single copy (LSC and SSC) regions (Fig 2) [21, 48]. The expansion or contraction of the two IR regions produces variation of the four junction regions and provides a valuable signal for phylogenetic analysis [48]. The dynamic variation in IR regions can cause the size changes of chloroplast genome. For example, previous studies have shown that the variation of the junctions in *Oryza* exceeds the junction variability in *Zizania* [15]. Between *C*. *mutica* and *C*. *aquatic*, no junction length variation was found with a similar result for the two *Zizania* species (Fig 2). Limited junction length variation between these groups indicates a conserved structure in the Zizaniinae subtribe. We also compared the dynamic variation of junctions between the Zizaniinae and Oryzinae subtribes (Fig 2).

For J_LA_, located in the intergenic region of *rps19*-*psbA*, the distances between *rps19* and J_LA_ varied in length from 41 bp to 49 bp and the distance between *psbA* and J_LA_ was from 81 bp to 83 bp in Oryzinae. In Zizaniinae, those distances were from 41 bp to 44 bp and 81 bp to 82 bp, respectively. For J_LB_, positioned between *rpl22* and *rps19*, the distances between *rpl22* and J_LB_ varied from 24 bp to 30 bp in Oryzinae, and in Zizaniinae, the distance was consistently 24 bp. From analysis of those two junctions, the variation in Oryzinae was greater than in Zizaniinae. However, the variability in distances for J_SA_ and J_SB_ were greater than J_LA_ and J_LB_. For J_SA_ in all species, the *ndhH* gene spanned this junction in the Oryzinae subtribe. The distance that the *ndhH* gene overlapped the junction, which varied from 163 bp to 625 bp in Oryzinae, while in Zizaniinae, the overlap was consistently 181 bp. For J_SB_, near the *ndhF* gene, the distance varied from 17 bp to 42 bp in Oryzinae but from 89 bp to 93 bp in Zizaniinae. The junction comparisons indicate that the structural variation in the Oryzinae subtribe varies more widely than in Zizaniinae. Furthermore, these junction comparisons indicate that J_LA_ and J_LB_ is less variable in length than J_SA_ and J_SB_, with the former less variable than the latter. From this, variations of J_SB_ could be used as molecular markers to separate the two subtribes given that the distance in Zizaniinae was twice as long as that in Oryzinae for J_SB_.

### Polymorphic variation

The two chloroplast genomes from *Chikusichloa* were found to be only 40 bp different in length with *C*. *mutica* shorter than *C*. *aquatica* (Table 2). In addition to total length differences, we assessed SNP and Indel variations between the entire chloroplast genomes of *C*. *mutica* and *C*. *aquatica* (Fig 1 and Table 4). In total, only 83 SNPs and 25 Indels were reported from the genome comparisons. For the SNPs, 58, 8 (16) and 9 were from LSC, IRs and SSC regions, respectively. For the 25 Indels, 21, 1(2) and 2 were within the LSC, IR and SSC regions. The distribution of these polymorphisms in the genome was as follows: 41, 8 (16) and 7 SNPs were from LSC, IR and SSC regions, and 20, 1(2) and 2 Indels were within LSC, IR and SSC regions, respectively. Most of the Indels and SNP variations were found from non-coding regions, including 64 SNPs and 24 Indels. Nineteen SNPs and 1 Indel were found in the coding regions, with the one Indel 21 base pairs into the *rps18* gene. Thirteen of those coding SNPs were as synonymous substitutions, and only six of them were as non- synonymous substitutions (S2 Table). Those six non-synonymous substitutions are also from just six different genes: *matK*, *rpoB*, *rpoC2*, *ndhJ*, *rpl16* and *ndhD*. The types of mutations between the two genomes were 41 transitions and 42 transversions among the 83 SNPs, and among the 25 Indels, 16 were homopolymer repeats, 4 repeat-related Indels and 5 independent Indels. Eleven of 16 homopolymer variations were A/T single repeats. This homopolymer variation is also consistent with previous findings [47].

**Table 4:** The number and distribution of polymorphisms of chloroplast genome between two *Chikusichloa* species.

Type A	Region	Coding Regions			Non-Coding Regions			Sum
SNP	LSC	17			41			58
	IR	0			16			16
	SSC	2			7			9
	Total	19			64			83
Type B	Region	Coding Regions			Non-Coding Regions			Sum
		Indel	Poly	Repeat	Indel	Poly	Repeat	
INDEL	LSC	0	0	1	2	16	2	21
	IR	0	0	0	2	0	0	2
	SSC	0	0	0	1	0	1	2
	Total	0	0	1	5	16	3	25

### Phylogeny

**Fig 3:**
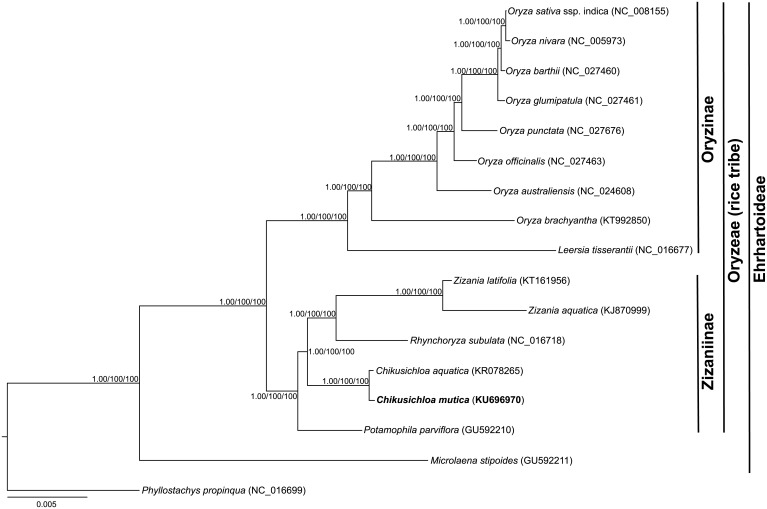
The chloroplast phylogenomic trees were generated from 17 grass species. Three different methods as Bayesian inference (BI), maximum parsimony (MP) and maximum likelihood (ML) were employed to build the tree. Numbers above the branches were the posterior probabilities for BI and bootstrap values of MP and NL. Branch length is proportional to the number of substitutions, as indicated by the scale bar.

The chloroplast genome has been widely used as an important source for molecular markers in plant systematics [49, 50]. However, with the development of high-throughput sequencing, the whole chloroplast genome has recently been used in phylogenetic studies as chloroplast phylogenomics [14, 19, 27]. The conserved structure among grass species chloroplast genomes has been reported from other lineages [14, 37] (S2 Fig). In this study, by employing the whole chloroplast genome alignment and three different methods to resolve the phylogenetic relationships among 16 species from the Ehrhartoideae subfamily and one bamboo species as an outgroup (Fig 3), two clades corresponding to the subtribes Oryzinae and Zizaniinae were resolved with high support (as 100 for ML and MP and 1.0 for BI). Within each clade, the relationships among species matched the topology of previous studies, which used partial chloroplast and/or nuclear genes [6, 34]. In subtribe Zizaniinae, the two species in *Chikusichloa*, *C*. *mutica* and *C*. *aquatica* were closely clustered together as sister species with equal branch lengths. The two species in *Zizania* were resolved on branches of different lengths. The differing branch lengths in the Oryzinae suggest heterogeneous evolutionary history between these clades with regard to chloroplast evolution.

## Discussion

In this study, by employing the traditional Sanger sequencing method, we completely sequenced the chloroplast genome of *Chikusichloa mutica*. As an important resource in rice germplasm, the complete chloroplast genome provides a valuable genetic resource for breeding and molecular analysis. Furthermore, the set of conserved primers used in this study could be widely employed in all rice tribe species, as well as Poaceae in general [14, 35]. The chloroplast genome of *C*. *mutica* is extremely conserved in structure compared with other published grass chloroplasts, with the gene content and number the same as other published chloroplast genomes [14, 15, 16, 51]. In comparison with the other species in *Chikusichloa*, *C*. *mutica* was found to have very limited variations (Fig 1) across the whole chloroplast genome.

### Sequencing and assembly strategy

Since the first two complete chloroplast genomes were reported from liverwort [52] and tobacco[53] in 1986, the knowledge of the organization and evolution of chloroplast genomes has increased rapidly. Currently, more than 1,000 fully sequenced chloroplast genomes have been deposited in the public database, brought about by the recent developments in NGS technologies [23] as well as innovations in bioinformatics algorithms for assembly [54]. However, the sequencing quality from the traditional Sanger sequencing remains higher than other NGS technologies. The traditional Sanger method of genome sequencing and assembly is more laborious and costly compared with the NGS method[22]. With the development of NGS and corresponding assembled methods, dozens or hundreds of chloroplast genomes could be completed in less time [55, 56]. However, the assembled quality of those genomes should be carefully scrutinized [22]. For example, using the Sanger method, Wu et al [22] sequenced one wild rice chloroplast genome and compared it with another published genome generated by a NGS short reads method. They found that the assembled chloroplast genomes were heterogeneous in coding and noncoding regions. Although NGS methods can produce high coverage for the assembled genome, some questions remain unresolved. For example, NGS data from short reads is difficult to assemble with regard to repeat regions across the genome [57]. Further complicating the solution to short read data is the fact that longer reads appear to possess more sequencing errors [58]. The traditional Sanger sequencing method is still one of the most effective ways to complete high quality genomes in spite of its higher cost and time investment compared to NGS methods. By employing this traditional Sanger method to complete a high-quality chloroplast genome for one wild rice—*C*. *mutica*, this study provided many valuable informative markers for future studies. However, with the new generation of sequencing technology, those high error rate sequencing could be improved lots and will change the way of sequencing. The third-generation genomic technologies have been widely used in many species [59, 60]. For example, the long-read sequencing technology from Pacific Biosciences’ Single Molecule Real-Time (SMRT) sequencing can generate reads with an average ~20 kb size, but the error of raw reads can be up to 15% [61]. However, if this SMRT technology could be combined with short sequencing reads as Illumina or by self-correction with sufficient sequencing data, the accuracy of the assembled genome can be improved to over 99.99%.

### Conserved chloroplast genome features in the grass family

The typical and stable quadripartite structure in chloroplast genomes, including a pair of IRs separating the LSC and SSC regions, has been reported in thousands of species [21, 26]. Among all published chloroplast genomes of the grass family, these conserved structures have been reported in all studies [14, 34, 37]. With regard to the genome size, the length variation of the whole chloroplast genome varies from 132 kb to 141 kb across Poaceae [14, 37]. In comparison, the SSC region is more stable in length than the LSC and IRs regions, with a length of approximately 12.5 kb. In contrast, the LSC region varies from 78.0 kb to 83.5 kb, and the IR region varies from 19.0 kb to 22.0 kb. The main reason for variation in genome length is expansions and contractions in the intergenic regions. For our sequenced *C*. *mutica*, the genome features are intermediate in length in relation to other Poaceae chloroplasts (Table 1). Secondly, the four junctions of the chloroplast genome [48] were consistently located in the same gene regions (Fig 2). Dynamic placement of junctions indicates the variation of the IR regions [21], and as such, the junction positions could be used in phylogenetic analyses [48]. For example, in *Chikusichloa*, the distances in all four junctions were the same, but they were different in other species (Fig 2). Thirdly, the gene content for all published chloroplast genomes in the grass family are the same as *C*. *mutica* (S1 Table). A total of 78 unique protein coding genes and 30 tRNA and 4rRNA genes were annotated among all grass species [14, 37]. All monocots have lost the *infA*, *accD*, *ycf*1 and *ycf*2 genes from their most recent common ancestors with dicots [62]. Although the conserved features of the chloroplast genome in the grass family are highly conserved, numerous microstructural variations (such as small insertions and deletions and SSR variation) have been found and constitute a valuable resource in phylogenetic and population analyses [22, 63]. The high-quality chloroplast genome of *C*. *mutica* reported here will be a valuable asset for discovering chloroplast variation in other Poaceae species.

### Limited variation within the *Chikusichloa* genus

Polymorphic markers in chloroplast genomes between different species have provided an abundance of informative loci in plant systematic or barcoding research [49, 64]-. In this study, we comprehensively compared the polymorphisms, including the SNPs and Indels, between the two fully sequenced chloroplast genomes of *C*. *mutica* (KU696970) and *C*. *aquatic* (KR078265). We found extremely limited variations, with only 83 SNPs and 24 Indels from the 136,640-bp alignment matrix between the two species. Most of the polymorphisms from coding genes are also synonymous, only six SNP from six genes are identified as non- synonymous. This also reflects that the variation of those polymorphisms is rare as adaptive. In contrast to *Chikusichloa*, in *Zizania*, 744 SNPs and 137 Indels were reported between *Z*. *latifolia* and *Z*. *aquatica* [15]. Several reasons might explain the differences found between the two genera. First, if the divergence times of *Zizania* were earlier than *Chikusichloa*, more variations could accumulate. However, the divergence times between the two genera were nearly equal at approximately 4 MYA [34]. Thus, differences in divergence times do not explain the differences in polymorphisms between the genera. Second, the distribution of species might drive the differences: all three species in genus *Chikusichloa* are located in Southeast Asia, whereas *Zizania* has a broad geographic distribution, with *Z*. *latifolia* and *Z*. *aquatica* separately distributed in Asia and North America [8]. The geographic patterns between these species, indicating a broad radiation and/or long-distance dispersal event, might explain the differences in polymorphisms. Partial lineage-specific variations from their own chloroplast genome were reflected the long distance of the segregation [25, 65]. This can be seen from the phylogenetic relationships (Fig 3): the branches of two *Chikusichloa* species are the same, while the branch lengths between the two *Zizania* species are longer. Several other factors could also cause such differences, such as the efficiency of the inner DNA polymerase, differences in the molecular evolutionary rate, and demographic history. Additional work is needed to clarify the causes of the different rates of polymorphism found in Zizaniinae.

## Conclusion

Using traditional high-quality Sanger sequencing technology, we presented the complete chloroplast genome of *Chikusichloa mutica*, performed comparative analyses in related species of the rice tribe, and deposited the genome into GenBank with accession number KU696970. The gene content, number and genome organization of *C*. *mutica* were identical to all other chloroplast genomes from Poaceae. From the whole genome comparison, limited variations were reported between two *Chikusichloa* species, with only 83 SNPs and 24 Indels between them. Phylogenetic analysis using whole genome sequences from 17 species in grass demonstrated the close relationship of two *Chikusichloa* species and also confirmed their phylogenetic position in relation to other rice tribe species. The full chloroplast genome data of *C*. *mutica* will facilitate the biological study of this important wild rice species. Furthermore, the chloroplast genome sequence is a valuable genetic resource that can be used to conduct population studies for this species and help shed light on its genetic mechanisms and evolutionary history.

## Supporting information

S1 Fig The full chloroplast reference genome of *Chikusichloa mutica*. The inside of the outer circle means the counterclockwise transcribed genes and the outside shows as the clockwise transcribed genes. Gray areas in the inner circle indicate the GC content as darker gray and the AT content as lighter gray. Genes belonging to different functional groups are color coded. LSC = large single copy; IR = inverted repeat; SSC = small single copy.
(TIF)

S2 Fig The whole chloroplast genome sequence identity plots containing two *Chikusichloa* species, two *Zizania* species with *O*. *sativa* ssp. Japonica (AY522330) as the reference genome. The vertical scale indicates the percentage of sequence identity (50%-100%). The horizontal axis shows the base position from the AY522330 chloroplast genome. Genome regions are color coded as protein-coding, rRNA, tRNA, intron, and conserved noncoding sequences (CNS) at bottom. The diagram was generated with mVISTA (http://genome.lbl.gov/vista/mvista/submit.shtml).
(EPS)

S1 File Whole chloroplast genome alignment of 17 species from grass family. (NEX)

S1 Table Gene content encoded in the *C*. *mutica* chloroplast genome. (DOCX)

S2 Table Polymorphic information from comparisons between two *Chikusichloa* species. (XLSX)

## References

[1] Grass Phylogeny Work. Group II. (2012) New grass phylogeny resolves deep evolutionary relationships and discovers C 4 origins. New Phytol 193:304–312. 10.1111/j.1469-8137.2011.03972.x 22115274

[2] Yu J, Hu S, Wang J, Wong GK, Li S, Liu B, et al (2002) A Draft Sequence of the Rice Genome (Oryza sativa L. ssp. indica). Science 296:79–92. 10.1126/science.1068037 11935017

[3] Ai B, Wang ZS, Ge S (2012) Genome size is not correlated with effective population size in the oryza species. Evolution (NY) 66:3302–3310. 10.1111/j.1558-5646.2012.01674.x 23025618

[4] Zou XH, Du YS, Tang L, Xu XW, Doyle JJ, Sang T, et al (2015) Multiple origins of BBCC allopolyploid species in the rice genus (Oryza). Sci Rep 5:14876 10.1038/srep14876 26460928 PMC4602239

[5] Guo YL, Ge S (2005) Molecular phylogeny of Oryzeae (Poaceae) based on DNA sequences from chloroplast, mitochondrial, and nuclear genomes. Am J Bot 92:1548–1558. 10.3732/ajb.92.9.1548 21646172

[6] Tang L, Zou X, Zhang L, Ge S (2015) Multilocus species tree analyses resolve the ancient radiation of the subtribe Zizaniinae (Poaceae). Mol Phylogenet Evol 84:232–239. 10.1016/j.ympev.2015.01.011 25655566

[7] Li ZM, Zheng XM, Ge S (2011) Genetic diversity and domestication history of African rice (Oryza glaberrima) as inferred from multiple gene sequences. Theor Appl Genet 123:21–31. 10.1007/s00122-011-1563-2 21400109

[8] Xu XW, Wu JW, Qi MX, Lu QX, Lee PF, Lutz S, et al (2015) Comparative phylogeography of the wild-rice genus Zizania (Poaceae) in eastern Asia and North America. Am J Bot 102:239–247. 10.3732/ajb.1400323 25667077

[9] Dong ZY, Wang YM, Zhang ZJ, Shen Y, Lin XY, Ou XF (2006) Extent and pattern of DNA methylation alteration in rice lines derived from introgressive hybridization of rice and Zizania latifolia Griseb. Theor Appl Genet 113:196–205. 10.1007/s00122-006-0286-2 16791687

[10] Eizenga GC, Agrama HA, Lee FN, Jia Y (2009) Exploring genetic diversity and potential novel disease resistance genes in a collection of rice (Oryza spp.) wild relatives. Genet Resour Crop Evol 56:65–76. 10.1007/s10722-008-9345-7

[11] Kim H, Hurwitz B, Yu Y, Collura K, Gill N, SanMiguel P, et al (2008) Construction, alignment and analysis of twelve framework physical maps that represent the ten genome types of the genus Oryza. Genome Biol 9:R45 10.1186/gb-2008-9-2-r45 18304353 PMC2374706

[12] Wang M, Yu Y, Haberer G, Marri PR, Fan C, Goicoechea JL, et al (2014) The genome sequence of African rice (Oryza glaberrima) and evidence for independent domestication. Nat Genet 46:982–988. 10.1038/ng.3044 25064006 PMC7036042

[13] Zhang QJ, Zhu T, Xia EH, Shi C, Liu YL, Zhang Y, et al (2014) Rapid diversification of five Oryza AA genomes associated with rice adaptation. Proc Natl Acad Sci U S A 111: E4954–E4962. 10.1073/pnas.1418307111 25368197 PMC4246335

[14] Wu ZQ, Ge S (2012) The phylogeny of the BEP clade in grasses revisited: Evidence from the whole-genome sequences of chloroplasts. Mol Phylogenet Evol 62:573–578. 10.1016/j.ympev.2011.10.019 22093967

[15] Wu ZQ, Gu C, Tembrock LR, Ge S (2015a) Limited Polymorphisms between Two Whole Plastid Genomes in the Genus Zizania (Zizaniinae). J Proteomics Bioinform 8:253–259. 10.4172/jpb.1000377

[16] Zhang J, Zhang D, Shi C, Gao J, Gao LZ (2016) The complete chloroplast genome sequence of *Chikusichloa aquatica* (Poaceae: Oryzeae). Mitochondrial DNA Part A 27:2771–2772. 10.3109/19401736.2015.1053058 26190082

[17] Wu ZY, Peter RH, Hong DY(2006) Flora of China, Volume 22: Poaceae.

[18] Howe CJ, Barbrook AC, Koumandou VL, Nisbet RE, Symington HA, Wightman TF (2003) Evolution of the chloroplast genome. Philos Trans R Soc Lond B Biol Sci 358:99-106-107. 10.1098/rstb.2002.1176 12594920 PMC1693101

[19] Gao L, Su YJ, Wang T (2010) Plastid genome sequencing, comparative genomics, and phylogenomics: Current status and prospects. J Syst Evol 48:77–93. 10.1111/j.1759-6831.2010.00071.x

[20] Neuhaus HE, Emes MJ (2000) Nonphotosynthetic Metabolism In Plastids. Annu Rev Plant Physiol Plant Mol Biol 51:111–140. 10.1146/annurev.arplant.51.1.111 15012188

[21] Ravi V, Khurana JP, Tyagi a. K, Khurana P (2008) An update on chloroplast genomes. Plant Syst Evol 271:101–122. 10.1007/s00606-007-0608-0

[22] Wu ZQ, Tembrock LR, Ge S (2015b) Are Differences in Genomic Data Sets due to True Biological Variants or Errors in Genome Assembly: An Example from Two Chloroplast Genomes. PLoS One 10:e0118019. 25658309 10.1371/journal.pone.0118019 PMC4320078

[23] Mardis ER (2013) Next-Generation Sequencing Platforms. Annu Rev Anal Chem 6:287–303. 10.1146/annurev-anchem-062012-092628 23560931

[24] Jansen RK, Raubeson LA, Boore JL, dePamphilis CW, Chumley TW, Haberle RC, et al (2005) Methods for Obtaining and Analyzing Whole Chloroplast Genome Sequences. Methods Enzymol 395: 348–384 10.1016/S0076-6879(05)95020-9 15865976

[25] Muse SV, Gaut BS (1997) Comparing patterns of nucleotide substitution rates among chloroplast loci using the relative ratio test. Genetics 146:393–399. 9136027 10.1093/genetics/146.1.393 PMC1207954

[26] Wicke S, Schneeweiss GM, dePamphilis CW, Müller KF, Quandt D (2011) The evolution of the plastid chromosome in land plants: gene content, gene order, gene function. Plant Mol Biol 76:273–297. 10.1007/s11103-011-9762-4 21424877 PMC3104136

[27] Jansen RK, Cai Z, Raubeson LA, Daniell H, Depamphilis CW, Leebens-Mack J, et al (2007) Analysis of 81 genes from 64 plastid genomes resolves relationships in angiosperms and identifies genome-scale evolutionary patterns. Proc Natl Acad Sci U S A 104:19369–19374. 10.1073/pnas.0709121104 18048330 PMC2148296

[28] Wang L, Qi XP, Xiang QP, Heinrichs J, Schneider H, Zhang XC (2010) Phylogeny of the paleotropical fern genus Lepisorus (Polypodiaceae, Polypodiopsida) inferred from four chloroplast DNA regions. Mol Phylogenet Evol 54:211–225. 10.1016/j.ympev.2009.08.032 19737617

[29] Wang L, Wu ZQ, Bystriakova N, Ansell SW, Xiang QP, Heinrichs J, et al (2011) Phylogeography of the Sino-Himalayan Fern Lepisorus clathratus on “the roof of the world”. PLoS One 6:e25896 10.1371/journal.pone.0025896 21984953 PMC3184171

[30] Dong W, Liu J, Yu J, Wang L, Zhou S (2012) Highly Variable Chloroplast Markers for Evaluating Plant Phylogeny at Low Taxonomic Levels and for DNA Barcoding. PLoS One 7:e35071 10.1371/journal.pone.0035071 22511980 PMC3325284

[31] DeGray G, Rajasekaran K, Smith F, Sanford J, Daniell H (2001) Expression of an antimicrobial peptide via the chloroplast genome to control phytopathogenic bacteria and fungi. Plant Physiol 127:852–862. 10.1104/pp.010233 11706168 PMC129257

[32] De Cosa B, Moar W, Lee SB, Miller M, Daniell H (2001) Overexpression of the Bt cry2Aa2 operon in chloroplasts leads to formation of insecticidal crystals. Nat Biotechnol 19:71–74. 10.1038/83559 11135556 PMC4560096

[33] Daniell H (2007) Transgene containment by maternal inheritance: effective or elusive? Proc Natl Acad Sci U S A 104:6879–6880. 10.1073/pnas.0702219104 17440039 PMC1855423

[34] Tang L, Zou XH, Achoundong G, Potgieter C, Second G, Zhang DY, et al (2010) Phylogeny and biogeography of the rice tribe (Oryzeae): Evidence from combined analysis of 20 chloroplast fragments. Mol Phylogenet Evol 54:266–277. 10.1016/j.ympev.2009.08.007 19683587

[35] Wu FH, Kan DP, Lee SB, Daniell H, Lee YW, Lin CC, et al (2009) Complete nucleotide sequence of Dendrocalamus latiflorus and Bambusa oldhamii chloroplast genomes. Tree Physiol 29:847–856. 10.1093/treephys/tpp015 19324693 PMC2762994

[36] Krzywinski M, Schein J, Birol I, Connors J, Gascoyne R, Horsman D, et al (2009) Circos: An information aesthetic for comparative genomics. Genome Res 19: 1639–1645. 10.1101/gr.092759.109 19541911 PMC2752132

[37] Zalapa JE, Cuevas H, Zhu H, Steffan S, Senalik D, Zeldin E, et al (2012) Using next-generation sequencing approaches to isolate simple sequence repeat (SSR) loci in the plant sciences. Am J Bot 99:193–208. 10.3732/ajb.1100394 22186186

[38] Katoh K, Standley DM (2013) MAFFT Multiple Sequence Alignment Software Version 7: Improvements in Performance and Usability. Mol Biol Evol 30:772–780. 10.1093/molbev/mst010 23329690 PMC3603318

[39] Librado P, Rozas J (2009) DnaSP v5: a software for comprehensive analysis of DNA polymorphism data. Bioinforma 25:1451–1452. 10.1093/bioinformatics/btp187 19346325

[40] Buschiazzo E, Gemmell NJ (2006) The rise, fall and renaissance of microsatellites in eukaryotic genomes. BioEssays 28:1040–1050. 10.1002/bies.20470 16998838

[41] Zalapa JE, Cuevas H, Zhu H, Steffan S, Senalik D, Zeldin E, et al (2012) Using next-generation sequencing approaches to isolate simple sequence repeat (SSR) loci in the plant sciences. Am J Bot 99:193–208. 10.3732/ajb.1100394 22186186

[42] Cotton JL, Wysocki WP, Clark LG, Kelchner SA, Pires JC, Edger PP, et al (2015) Resolving deep relationships of PACMAD grasses: a phylogenomic approach. BMC Plant Biol 15:178 10.1186/s12870-015-0563-9 26160195 PMC4498559

[43] Swofford DL (2002) PAUP*: Phylogenetic Analysis Using Parsimony (and other methods).

[44] Ronquist F, Teslenko M, van der Mark P, Ayres DL, Darling A, Höhna S, et al (2012) MrBayes 3.2: Efficient Bayesian Phylogenetic Inference and Model Choice across a Large Model Space. Syst Biol 10.1093/sysbio/sys029 22357727 PMC3329765

[45] Guindon S, Gascuel O (2003) A simple, fast, and accurate algorithm to estimate large phylogenies by maximum likelihood. Syst Biol 52:696–704. 10.1080/10635150390235520 14530136

[46] Michelangeli FA, Davis JI, Stevenson DW (2003) Phylogenetic relationships among Poaceae and related families as inferred from morphology, inversions in the plastid genome, and sequence data from the mitochondrial and plastid genomes. Am J Bot 90:93–106. 10.3732/ajb.90.1.93 21659084

[47] Kuang DY, Wu H, Wang YL, Gao LM, Zhang SZ, Lu L (2011) Complete chloroplast genome sequence of Magnolia kwangsiensis (Magnoliaceae): implication for DNA barcoding and population genetics. Genome 54:663–673. 10.1139/G11-026 21793699

[48] Wang RJ, Cheng CL, Chang CC, Wu CL, Su TM, Chaw SM (2008) Dynamics and evolution of the inverted repeat-large single copy junctions in the chloroplast genomes of monocots. BMC Evol Biol 8:36 10.1186/1471-2148-8-36 18237435 PMC2275221

[49] Shaw J, Lickey EB, Beck JT, Farmer SB, Liu W, Miller J, et al (2005) The tortoise and the hare II: relative utility of 21 noncoding chloroplast DNA sequences for phylogenetic analysis. Am J Bot 92:142–166. 10.3732/ajb.92.1.142 21652394

[50] Shaw J, Shafer HL, Leonard OR, Kovach MJ, Schorr M, Morris AB (2014) Chloroplast DNA sequence utility for the lowest phylogenetic and phylogeographic inferences in angiosperms: The tortoise and the hare IV. Am J Bot 101:1987–2004. 10.3732/ajb.1400398 25366863

[51] Saski C, Lee SB, Fjellheim S, Guda C, Jansen RK, Luo H, et al (2007) Complete chloroplast genome sequences of Hordeum vulgare, Sorghum bicolor and Agrostis stolonifera, and comparative analyses with other grass genomes. Theor Appl Genet 115:571–590. 10.1007/s00122-007-0567-4 17534593 PMC2674615

[52] Ohyama K, Fukuzawa H, Kohchi T, Shirai H, Sano T, Sano S, et al (1986) Chloroplast gene organization deduced from complete sequence of liverwort Marchantia polymorpha chloroplast DNA. Nature 322:572–574.

[53] Shinozaki K, Ohme M, Tanaka M, Wakasugi T, Hayashida N, Matsubayashi T, et al (1986) The complete nucleotide sequence of the tobacco chloroplast genome: its gene organization and expression. EMBO J 5:2043–2049. 16453699 10.1002/j.1460-2075.1986.tb04464.x PMC1167080

[54] Pabinger S, Dander A, Fischer M, Snajder R, Sperk M, Efremova M, et al (2014) A survey of tools for variant analysis of next-generation genome sequencing data. Brief Bioinform 15:256–278.: 10.1093/bib/bbs086 23341494 PMC3956068

[55] Cronn R, Liston A, Parks M, Gernandt DS, Shen R, Mockler T (2008) Multiplex sequencing of plant chloroplast genomes using Solexa sequencing-by-synthesis technology. Nucleic Acids Res 36:e122 10.1093/nar/gkn502 18753151 PMC2577356

[56] Bayly MJ, Rigault P, Spokevicius A, Ladiges PY, Ades PK, Anderson C, et al (2013) Chloroplast genome analysis of Australian eucalypts—Eucalyptus, Corymbia, Angophora, Allosyncarpia and Stockwellia (Myrtaceae). Mol Phylogenet Evol 69:704–716. 10.1016/j.ympev.2013.07.006 23876290

[57] Miller JR, Koren S, Sutton G (2010) Assembly algorithms for next-generation sequencing data. Genomics 95:315–327. 10.1016/j.ygeno.2010.03.001 20211242 PMC2874646

[58] Quail MA, Smith M, Coupland P, Otto TD, Harris SR, Connor TR, et al (2012) A tale of three next generation sequencing platforms: comparison of Ion torrent, pacific biosciences and illumina MiSeq sequencers. BMC Genomics 13:1. 22827831 10.1186/1471-2164-13-341 PMC3431227

[59] Berlin K, Koren S, Chin CS, Drake JP, Landolin JM, Phillippy AM (2015) Assembling large genomes with single-molecule sequencing and locality-sensitive hashing. Nat Biotechnol 33:623–630. 10.1038/nbt.3238 26006009

[60] VanBuren R, Bryant D, Edger PP, Tang H, Burgess D, Challabathula D, et al (2015) Single-molecule sequencing of the desiccation-tolerant grass Oropetium thomaeum. Nature 527:508–511. 10.1038/nature15714 26560029

[61] Jiao WB, Schneeberger K (2017) The impact of third generation genomic technologies on plant genome assembly. Curr Opin Plant Biol 36:64–70. 10.1016/j.pbi.2017.02.002 28231512

[62] Guisinger MM, Chumley TW, Kuehl JV, Boore JL, Jansen RK (2010) Implications of the plastid genome sequence of typha (Typhaceae, Poales) for understanding genome evolution in poaceae. J Mol Evol 70:149–166. 10.1007/s00239-009-9317-3 20091301 PMC2825539

[63] Diekmann K, Hodkinson TR, Barth S (2012) New chloroplast microsatellite markers suitable for assessing genetic diversity of Lolium perenne and other related grass species. Ann Bot 110:1327–1339. 10.1093/aob/mcs044 22419761 PMC3478042

[64] CPBOL G, Li DZ, Gao LM, Li HT, Wang H, Ge XJ, et al (2011) Comparative analysis of a large dataset indicates that internal transcribed spacer (ITS) should be incorporated into the core barcode for seed plants. Proc Natl Acad Sci U S A 108:19641–19646. 10.1073/pnas.1104551108 22100737 PMC3241788

[65] Clegg MT, Gautt BS, Learn GH, Morton BR (1994) Rates and patterns of chloroplast DNA evolution. Proc Natl Acad Sci U S A 91:6795–6801. 8041699 10.1073/pnas.91.15.6795 PMC44285

