# Supplementary material for: Characterization of the whole chloroplast genome of Chikusichloa mutica and its comparison with other rice tribe (Oryzeae) species
Source: PLoS One. 2017 May 24;12(5):e0177553. doi: 10.1371/journal.pone.0177553 (PMC5443529; doi:10.1371/journal.pone.0177553)
Supplement: S1 Table — (DOCX) [file pone.0177553.s004.docx]

**Gene content encoded in the *C. mutica* chloroplast genome.**

| Group | Gene product | Number | Gene name |
| --- | --- | --- | --- |
| 1 | Photosystem I | 5 | *psa*A; B; C; I; J; |
| 2 | Photosystem II | 15 | *psb*A; B; C; D; E; F; H; I; J; K; L; M; N; T; Z |
| 3 | Cytochromeb6/f | 6 | *pet*A; B^a^; D^a^; G; L; N |
| 4 | ATP synthase | 6 | *atp*A; B; E; F^a^; H; I |
| 5 | Rubisco | 1 | *rbcL;* |
| 6 | NADH oxidoreductase | 11 | *ndh*A^a^; B^a, c^; C; D; E; F; G; H; I; J; K |
| 7 | ribosomal proteins (LSU) | 9 | *rpl2*^a,c^ ;14;16^a^; 20; 22; 23^c^; 32; 33; 36 |
| 8 | ribosomal proteins (SSU) | 12 | *rps*2; 3; 4; 7^c^; 8; 11; 12^a, c, d^; 14; 15; 16^a^; 18; 19^c^ |
| 9 | RNA polymerase | 4 | *rpoA; rpoB; rpoC1; rpoC2* |
| 10 | Other proteins | 5 | *ccsA; cemA; clpP; infA; matK* |
| 11 | Proteins of unknown function | 2 | *ycf3*^b^; *ycf*4 |
| 12 | Ribosomal RNAs | 4 | *rrn4*.5^c^; 5^c^; 16^c^; 23^c^ |
| 13 | Transfer RNAs | 30 | trnH(GUG)^c^; K(UUU)^a^; Q(UUG); S(GCU); G(UCC)^a^; R(UCU); C(GCA); D(GUC); Y(GUA); E(UUC); T(GGU); S(UGA); G(GCC); fM(CAU); S(GGA); T(UGU); L(UAA)^a^; F(GAA); V(UAC)^a^; M(CAU); W(CCA); P(UGG); I(CAU)^c^; L(CAA) ^c^; V(GAC) ^c;^ I(GAU)^a,c^; A(UGC)^a,c^ ; R(ACG) ^c;^ N(GUU) ^c^; L(UAG) |

a: Gene containing a single intron;

b: Gene containing two introns;

c: Two copies in the IRs;

d: Gene containing trans-splicing introns;
